# Seasonal Variation and Factors Affecting *Trypanosoma theileri* Infection in Wild Sika Deer (Ezo Sika Deer *Cervus nippon yesoensis*) in Eastern Hokkaido

**DOI:** 10.3390/ani13101707

**Published:** 2023-05-22

**Authors:** Yujon Hong, Keisuke Suganuma, Yuma Ohari, Mitsunori Kayano, Kenji Nakazaki, Shinya Fukumoto, Shin-ichiro Kawazu, Noboru Inoue

**Affiliations:** 1 National Research Center for Protozoan Diseases, Obihiro University of Agriculture and Veterinary Medicine, Inada, Obihiro 080-8555, Hokkaido, Japan; yt9171@gmail.com (Y.H.); whale15191670@gmail.com (K.N.); fukumoto@obihiro.ac.jp (S.F.); skawazu@obihiro.ac.jp (S.-i.K.); ircpmi@obihiro.ac.jp (N.I.); 2 Research Center for Global Agromedicine, Obihiro University of Agriculture and Veterinary Medicine, Inada, Obihiro 080-8555, Hokkaido, Japan; kayano@obihiro.ac.jp; 3 Division of Risk Analysis and Management, International Institute for Zoonosis Control, Hokkaido University, Kita 20 Jyo Nishi 10, Sapporo 001-0020, Hokkaido, Japan; y_ohari@czc.hokudai.ac.jp

**Keywords:** Ezo sika deer, *Megatrypanum*, risk factors, seasonal variation, *Trypanosoma*

## Abstract

**Simple Summary:**

The prevalence of parasitic infection exhibits seasonal variation and is affected by several factors, including environmental and host conditions. In this study, we investigated the seasonal variation in and factors affecting *Trypanosoma theileri* Laveran, 1902, infection in wild sika deer (Ezo sika deer) *Cervus nippon yesoensis* (Heude, 1884) in Eastern Hokkaido, Japan. The seasonal prevalence of trypanosome infection ranged from 0 to 41% (mean: 13%) based on hematocrit concentrations and from 17 to 89% (mean: 46%) based on PCR results. The factors identified as significantly affecting trypanosome infection included host age and sampling season, as per multiple logistic regression analysis. This is the first study to investigate the seasonal variation of and risk factors affecting trypanosome infection in wild deer.

**Abstract:**

*Trypanosoma* (*Megatrypanum*) spp. are isolated from domestic and wild ruminants, including deer, worldwide. The prevalence of trypanosomes in mammals is influenced by a number of factors such as host age and vector abundance. However, the seasonal variation of and factors affecting trypanosome infection in the wild deer population remain elusive. In this study, we analyzed the seasonal variation in trypanosome prevalence and the factors that affect *Trypanosoma theileri* Laveran, 1902, infection in wild sika deer (Ezo sika deer) *Cervus nippon yesoensis* (Heude, 1884) in Eastern Hokkaido through a two-year survey. Seasonal variation in the prevalence of trypanosome infection in the deer population ranged from 0 to 41% as per hematocrit concentration and 17 to 89% as per PCR results. In general, the prevalence of *T. theileri* by PCR in 2020 was higher than that in 2019. Moreover, the prevalence was significantly higher in the aged population than among the younger population. These findings may explain why individual conditions and sampling season were associated with trypanosome prevalence. This is the first study to investigate the seasonal variation in and risk factors affecting trypanosome infection in wild deer.

## 1. Introduction

Various *Megatrypanum* trypanosomes have been isolated from domestic and wild ruminants, such as cattle, water buffaloes, and deer. *Trypanosoma* (*Megatrypanum*) spp. infections in Cervidae have been reported in the caribou *Rangifer tarandus caribou* (Gmelin, 1788) [1], roe deer *Capreolus capreolus* (Linnaeus, 1758) [2], reindeer *Rangifer tarandus* (Linnaeus, 1758) [3], mule deer *Odocoileus hemionus* (Rafinesque, 1817) [4,5,6], moose *Alces alces* (Linnaeus, 1758), white-tailed deer *Odocoileus virginianus* (Zimmerman, 1780) [7,8,9], and elk *Cervus elaphus canadensis* (Eexleben, 1777) [7] in North and South America [10]. In Europe, infections have been reported in the fallow deer *Cervus dama* Linnaeus, 1758, red deer *Cervus elaphus* Linnaeus, 1758, and roe deer in Germany [11]; reindeer and moose in Sweden [12]; and in roe deer, red deer, and elk in Poland [13,14,15,16]. In Japan, *T.* (*Megatrypanum*) spp., named the TSD1 isolate, was isolated from wild sika deer (Ezo sika deer) *Cervus nippon yesoensis* (Heude, 1884) and was distinguished from *T. theileri* Laveran, 1902, based on differences in karyotype determined via pulsed-field gel electrophoresis [17]. In addition, genotypically distinct *T. theileri* isolates were detected in Honshu sika deer *Cervus nippon centralis* Temminck, 1836 [18].

Several factors, such as sex, age, and breed, have been reported to affect trypanosome infection in domestic animals in previous studies [19,20,21]. Moreover, the seasonal variation in trypanosome infection is strongly correlated with that of its vector insects [22,23,24]. However, no studies have addressed the seasonal variation of *T. theileri* prevalence nor the factors that affect trypanosome infection in the wild deer population because of the difficulty associated with continuous sampling. Therefore, we aimed to determine the seasonal variation of and the factors that affect *T. theileri* infection in Ezo sika deer, utilizing a combination of parasitological and molecular techniques.

## 2. Materials and Methods

### 2.1. Blood Sample Collection

**Figure 1:**
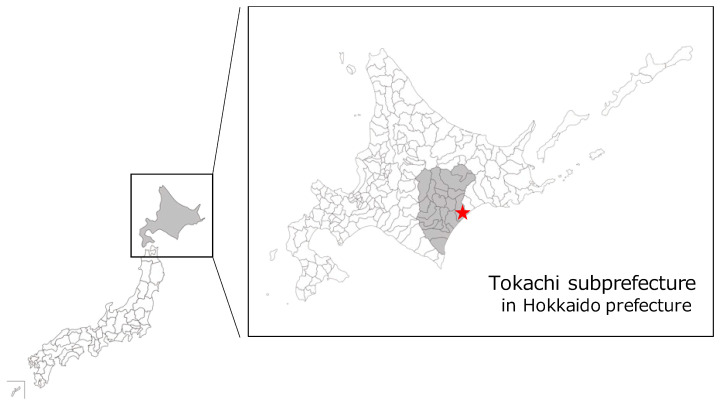
Map of the study area. The star symbol depicts the location of ELEZO company in Toyokoro-cho town.

From October 2019 to September 2021, 765 blood samples collected from Ezo sika deer were provided by the ELEZO company (Toyokoro-cho town, Hokkaido, Japan). All Ezo sika deer were commercially hunted in the Tokachi subprefecture, Hokkaido, Japan (Figure 1). The ELEZO company hunters acted in accordance with the guidelines published by the Ministry of the Environment and the Ministry of Agriculture, Forestry, and Fisheries of Japan. The hunters recorded the sex and age of deer, as well as the date and location of the hunting event. The age of deer was determined based on horn morphology for males and body constitution in maternal line clusters for females. In addition, if there were any characteristic clinical findings in the hunted deer, this information was also recorded. The blood samples were collected in centrifuge tubes with an anticoagulant (EDTA; Sigma-Aldrich Japan, Tokyo, Japan) and stored at 4 °C until parasitological examination. The present study was approved by the Committee on the Ethics of Animal Experiments of the Obihiro University of Agriculture and Veterinary Medicine (Approval number: 21-59).

### 2.2. Parasitological Examination

Microscopic observations of *T. theileri* in deer blood were conducted using the hematocrit concentration technique (HCT) [25]. Briefly, 60 µL of blood in hematocrit (Hematocrit capillaries Na-hep, HIRSCHMAN, Eberstadt, Germany) was centrifuged, and trypanosomes were concentrated in the buffy coat layer. After centrifugation, actively moving live trypanosomes in the upper layer of the buffy coat were detected using phase-contrast microscopy.

### 2.3. DNA Extraction and PCR

DNA was extracted from 200 µL of whole blood samples using the QIAamp Blood Mini kit (Qiagen, Hilden, Germany), according to the manufacturer’s instructions. The cathepsin L-like protein gene (*CATL*) was amplified via PCR from DNA samples to screen for *T. theileri* using *Megatrypanum* trypanosome-specific partial *CATL* primers (TthCATL1: 5′-CGT CTC TGG CTC CGG TCA AAC-3′ and rDTO155: 5′-TTA AAG CTT CCA CGA GTT CTT GAT CCA GTA-3′) [26].

A reaction mixture (10 μL) that contained 1 µL of the DNA sample, 5 µL of 2× MightyAmp buffer Ver. 3 (Takara Bio Inc., Shiga, Japan), 0.3 µM of each forward and reverse primer, 1 µL of 10× Additive for High Specificity (Takara Bio Inc.), 0.2 µL of MightyAmp DNA polymerase Ver. 3 (Takara Bio Inc.), and 2.2 μL of distilled water was prepared for each PCR assay. The PCR cycling conditions were as follows, as per the manufacturer’s protocol: initial pre-denaturation step at 98 °C for 2 min, 40 cycles of denaturation at 98 °C for 10 s, annealing at 63 °C for 15 s, and extension at 68 °C for 10 s. 

DNA extracted from the *T. theileri* Obihiro strain [27] was used as a positive control for the PCR, while distilled water was used as a negative control.

### 2.4. Data Analysis

Relationships between the prevalence of *T. theileri* and year (2019 and 2020), sex (female, male), age (1, 2, and ≥3 years old), and season (spring, summer, autumn, and winter) were assessed via univariate testing with chi-square tests. In addition, multivariate logistic regression was applied to the data set, excluding data that were not available (NA), with sex, age, and seasons as explanatory variables. In addition, to reveal inter-seasonal variation in the prevalence over two years, risk factors were separately analyzed in 2019 and 2020.

Because the hunting season in the study area lasted from October to March, the sampling year was planned from the start of the hunting season to the next hunting season (year of 2019: October 2019 to September 2020, year of 2020: from October 2020 to September 2021). In addition, the terminology for each season (spring, March to May; summer, June to August; autumn, September to November; and winter, December to February) was assigned in accordance with the definition provided by the Japan Meteorological Agency. All analyses were conducted using the R statistical software (version 4.0.4 for macOS).

## 3. Results

**Figure 2:**
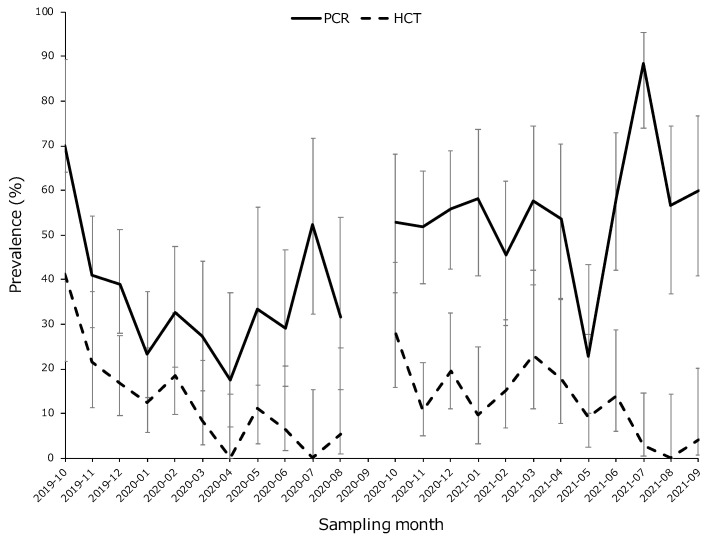
Seasonal variation in trypanosome infection prevalence from October 2019 to September 2021. The solid and dotted lines indicate the prevalence determined via PCR and hematocrit centrifugation technique (HCT), respectively. Each plot shows prevalence with a 95% confidence interval. No samples were obtained in September 2020 (2020-09).

None of the characteristic clinical symptoms were recorded in hunted deer. The overall prevalence (percentage of hosts infected) of *T. theileri* infection in Ezo sika deer was 13.42% (102/760), as determined via the HCT, and 45.62% (349/765), as determined through PCR. The monthly prevalence of *T. theileri* infection in Ezo sika deer from October 2019 to September 2021 is shown in Figure 2. 

In the results of HCT analysis, only one factor (season) was significantly associated with the prevalence of *T. theileri*. Prevalence was significantly different between seasons (*p* < 0.01): spring 11.76% (18/153), summer 5.45% (9/165), autumn 18.82% (32/170), and winter 15.81% (43/272) (Table 1). 

In the PCR results, all factors were significantly associated with the prevalence of *T. theileri* (Table 1). The prevalence in 2020 (56%, 224/400) was significantly higher than that in 2019 (34.25%, 125/365) (*p* < 0.01). Further, *T. theileri* prevalence was significantly different between seasons (*p* < 0.01): spring 36.00% (54/150), summer 55.15% (91/165), autumn 51.10% (93/182), and winter 41.42% (111/268). The prevalence in male deer (49.10%, 218/439) was significantly higher than that in female deer (40.81%, 131/321) (*p* = 0.03). In addition, age-wise comparison revealed that a higher prevalence of *T. theileri* was recorded in the old (2 years old: 47.13% (115/244) and over 3-year-old deer population: 51.44% (196/381)) than in the young population (1 year old: 27.14% (38/140)), with a significant difference (*p* < 0.01).

A summary of the results of multivariate logistic regression analysis is presented in Table 2, which also revealed that season significantly affected the prevalence of *T. theileri* based on the HCT. The deer population exhibited a more than two-fold lower prevalence in summer (odds ratio (OR) = 0.42, 95% confidence interval (CI) = 0.17−0.95, *p* = 0.04) than in spring. The same analysis based on PCR data revealed that year, season, and age significantly affected the prevalence of *T. theileri*. The deer population exhibited a more than two-fold higher prevalence in 2020 (OR = 2.34, 95% CI = 1.71–3.21, *p* < 0.01) than in 2019. In addition, the prevalence in summer (OR = 1.98, 95% CI = 1.23–3.21, *p* < 0.01) and autumn (OR = 1.64, 95% CI = 1.01–2.67, *p* = 0.04) was significantly higher than that in spring. The prevalence in older populations was over two times greater than (2 years old: OR = 2.64, 95% CI = 1.65–4.32, *p* < 0.01, and ≥ 3 years old: OR = 2.78, 95% CI = 1.77–4.43, *p* < 0.01) that in the young population (1 year old).

In addition, the association of risk factors in each year is summarized in Table 3. 

Based on HCT data, the prevalence of *T. theileri* was significantly higher during autumn in 2019 (OR = 4.97, 95% CI = 1.70–16.80, *p* < 0.01) but not in 2020 (OR = 0.85, 95% CI = 0.39–1.93, *p* = 0.70). Meanwhile, it was significantly lower in the summer of 2020 (OR = 0.32, 95% CI = 0.11–0.86, *p* = 0.03) but not in that of 2019 (OR = 0.52, 95% CI = 0.10–2.23, *p* = 0.39). In addition, significant differences based on deer age were observed in 2019, but not in 2020. No significant differences in *T. theileri* prevalence were observed between the remaining factors.

Based on PCR results, the prevalence of *T. theileri* was also significantly higher in the autumn of 2019 (OR = 2.61, 95% CI = 1.19–5.82, *p* = 0.02) but not in that of 2020 (OR = 1.28, 95% CI = 0.71–2.34, *p* = 0.41). Meanwhile, the prevalence was significantly higher in the summer of 2020 (OR = 2.49, 95% CI = 1.31–4.80, *p* < 0.01) but not in that of 2019 (OR = 1.52, 95% CI = 0.74–3.19, *p* = 0.26). Moreover, the prevalence in older populations was more than two times greater (2 years old in 2019: OR = 2.21, 95% CI = 1.07–4.83, *p* < 0.01 and ≥3 years old in 2019: OR = 2.91, 95% CI = 1.47–6.16, *p* < 0.01; 2 years old in 2020: OR = 3.30, 95% CI = 1.76–6.33, *p* < 0.01 and ≥3 years old in 2020: OR = 2.72, 95% CI = 1.51–5.02, *p* < 0.01) than that in the young population (1-year-olds) for both years. No significant differences were observed in the prevalence of *T. theileri* based on the remaining factors.

## 4. Discussion

In the present study, we analyzed the seasonal variation in *T. theileri* prevalence in Ezo sika deer in eastern Hokkaido and the factors that affect it. As no clinical symptoms were recorded, the target population of deer could be considered as apparently healthy. Overall, the prevalence evaluated using PCR (45.62%) was higher than that evaluated using the HCT (13.42%). This confirmed the findings of other studies, namely, that the PCR detection limit for trypanosomes is generally higher than that of the HCT [28,29,30,31,32].

Based on HCT data, only the sampling season had a significant effect on the prevalence. Meanwhile, univariate analysis based on the PCR data suggested that sampling year, season, sex, and age all significantly affected the prevalence of *T. theileri*. As per multivariate logistic regression analysis, sampling year, season, and age significantly affected trypanosome prevalence and detection rate.

The sampling season significantly affected the prevalence determined using either method. When using the HCT method, the prevalence in summer was significantly lower than that in spring. On the other hand, the prevalence in summer and autumn was significantly higher than that in spring as per PCR data. The study area (Hokkaido Prefecture) is located in the subarctic zone. From winter to early spring, heavy snow limits the adequate nutrition of wild deer populations. In summer, wild deer could sufficiently feed on grasses, and their body condition was better than that in winter [33].

Seasonal variation in the blood-sucking insect vectors could also influence the seasonal pattern of trypanosome infection prevalence. Horse flies and deer flies (*Tabanus* spp., *Haematopota* spp., *Chrysops* spp., and *Atylotus* spp.) were observed from the end of May to early October in Hokkaido Prefecture [34,35]. They feed on not only domestic animals but also wild deer [36]. These previous reports may suggest that the increased prevalence of *T. theileri* infection in wild deer populations during summer may be due to the role of blood-sucking horse flies as biological vectors, but the number of trypanosomes was still lower than the detection limit of HCT because infection by trypanosomes is possibly latent in healthy wild deer during the summer. Previous reports revealed that horse and deer flies act as biological vectors of *T. theileri* [37,38,39]. However, only one previous report has suggested a horsefly species (*Tabanus rufidens* (Bigot, 1887)) as a potential *T. theileri* vector in Japan [40]. While we have not yet confirmed the biological vectors of *T. theileri* in Ezo sika deer and domestic cattle within the study area, *T. theileri* may be transmitted via horse flies, and the seasonal variation in the number of horseflies could affect the respective prevalence of *T. theileri* in Ezo sika deer.

The sampling year could also explain the difference in the prevalence of trypanosome infection in Ezo sika deer. In general, the prevalence of *T. theileri* in Ezo sika deer within the study area was higher in 2020 than in 2019. As previously mentioned, the prevalence of trypanosome infection is possibly affected by the seasonal pattern of their vectors and the bodily condition of the host. Several studies showed that horsefly activity and abundance are negatively affected by considerable rainfall and low temperatures [41,42]. According to the meteorological data from a representative point within the study area (Automated Meteorological Data Acquisition System in Urahoro), provided by the Japan Meteorological Agency, the total rainfall from July to September 2019 (420 mm), which is the season of horsefly occurrence in the region, was greater than that in 2020 (236.5 mm). The blood-sucking behavior of horse flies was possibly suppressed by the higher amount of rainfall in 2019, in turn reducing *T. theileri* prevalence. In addition, heavy snowfall may also explain the higher prevalence of *T. theileri*, as it is associated with the undernutrition of deer resulting from poor food intake. The total snowfall during the winter of 2020 (December 2020 to February 2021; 183 cm) was greater than that in 2019 (December 2019 to February 2020; 104 cm). In addition, the heavy snowfall led to a considerable accumulation of snow in the field. Taken together, the combination of several environmental factors may underpin differences in the prevalence of *T. theileri* infection in Ezo sika deer. To confirm this notion, we should study trypanosome vectors in greater detail in our future research. Further, we shall continue sampling and analyzing deer trypanosome prevalence for several years, in a bid to determine the association of prevalence with climatic conditions.

The age of host deer was also revealed as a factor that significantly affects *T. theileri* prevalence based on PCR data, for both years. *T. theileri* infection possibly has a minor effect or is non-pathogenic in wild deer, as no clinical symptoms were recorded during sampling, and *T. theileri* is generally considered a low-impact pathogenic trypanosome in cattle [43], despite a marginal effect of trypanosome infection on dairy cattle productivity [44]. The older deer population had a higher risk of trypanosome infection via blood-sucking horseflies than the younger population. After the initial infection, trypanosomes usually parasitize through latent infections below the detection limit of the HCT, without symptoms. In addition, the older population had a greater chance of trypanosome transmission than the younger population.

In this study, we did not include sampling locations in the risk factor analysis because wild sika deer migrate over relatively large areas during their lifetime [45]. Therefore, the hunting location is expected not to be related to the prevalence of trypanosome infection. Previous reports on the spatial genetic structure of the Ezo sika deer population revealed three to four sub-populations [46]. Depending on the deer population and their local migration, the seasonal variation and factors affecting the prevalence of *T. theileri* infection could explain trypanosome infection rates in Ezo sika deer inhabiting the East Hokkaido area.

## 5. Conclusions

Herein, we determined the seasonal variation in *T. theileri* prevalence in Ezo sika deer. In addition, environmental (sampling year and sampling season) and individual (age of the deer) conditions affected the prevalence of *T. theileri* in Ezo sika deer. This is the first study on the seasonal variation in trypanosome infection prevalence in wild deer. 

## Figures and Tables

**Table 1:** Results of univariate analysis of risk factors associated with the prevalence of *T. theileri*.

	Factor						
Methods	Year	No. of samples	No. of positive samples	Positive %	[95% CI]	chi-square value	*p*-value
HCT	2019	359	48	13.37	[10.23–17.28]	<0.01	1
	2020	401	54	13.47	[10.47–17.16]		
PCR	2019	365	125	34.25	[29.56–39.26]	35.53	<0.01 *
	2020	400	224	56.00	[51.10–60.78]		
	Season	No. of samples	No. of positive samples	Positive %	[95% CI]	chi-square value	*p*-value
HCT	Spring	153	18	11.76	[7.57–17.83]	14.98	<0.01 *
	Summer	165	9	5.45	[2.90–10.04]		
	Autumn	170	32	18.82	[13.66–25.36]		
	Winter	272	43	15.81	[11.95–20.62]		
PCR	Spring	150	54	36.00	[28.76–43.94]	15.75	<0.01*
	Summer	165	91	55.15	[47.53–62.54]		
	Autumn	182	93	51.10	[43.89–58.26]		
	Winter	268	111	41.42	[35.68–47.40]		
	Sex	No. of samples	No. of positive samples	Positive %	[95% CI]	chi-square value	*p*-value
HCT	Female	439	62	14.12	[11.18–17.69]	0.31	0.58
	Male	321	40	12.46	[9.29–16.52]		
PCR	Female	444	218	49.10	[44.48–53.74]	4.83	0.03 *
	Male	321	131	40.81	[35.57–46.26]		
	Age	No. of samples	No. of positive samples	Positive %	[95% CI]	chi-square value	*p*-value
HCT	1	136	14	10.29	[6.23–16.54]	2.25	0.32
	2	242	38	15.70	[11.66–20.82]		
	≥3	381	50	13.12	[10.10–16.89]		
PCR	1	140	38	27.14	[20.46–35.05]	24.7	<0.01 *
	2	244	115	47.13	[40.96–53.39]		
	≥3	381	196	51.44	[46.44–56.42]		

* indicates significant differences (*p* < 0.05) between categories.

**Table 2:** Summary of the results from multivariate logistic regression analysis of risk factors associated with the prevalence of *T. theileri*.

Method	Factor	Category	OR	[95% CI]	*p*-Value
HCT	Year	2019	1 (Reference)		
		2020	1.24	[0.79–1.96]	0.35
	Season	Spring			Reference
		Summer	0.42	[0.17–0.95]	0.04 *
		Autumn	1.27	[0.66–2.49]	0.48
		Winter	0.95	[0.52–1.80]	0.88
	Sex	Female			Reference
		Male	0.69	[0.43–1.10]	0.12
	Age	1			Reference
		2	1.71	[0.89–3.46]	0.12
		≥3	1.2	[0.64–2.39]	0.58
PCR	Year	2019			Reference
		2020	2.34	[1.71–3.21]	<0.01 *
	Season	Spring			Reference
		Summer	1.98	[1.23–3.2]	<0.01 *
		Autumn	1.64	[1.01–2.67]	0.04 *
		Winter	1.34	[0.86–2.09]	0.21
	Sex	Female			Reference
		Male	0.79	[0.56–1.1]	0.16
	Age	1			Reference
		2	2.64	[1.65–4.32]	<0.01 *
		≥3	2.78	[1.77–4.43]	<0.01 *

* indicates significant differences (*p* < 0.05) compared with the reference categories.

**Table 3:** Results of multivariate logistic regression analysis of risk factors associated with the prevalence of *T. theileri* in 2019 and 2020.

**Sampling Year 2019 (October 2019–September 2020)**					
**Method**	**Factor**	**Category**	**OR**	**[95% CI]**	***p*-Value**
HCT	Season	Spring	1 (Reference)		
		Summer	0.52	[0.10–2.23]	0.39
		Autumn	4.97	[1.70–16.80]	<0.01 *
		Winter	2.35	[0.91–7.27]	0.1
	Sex	Female			Reference
		Male	0.65	[0.31–1.3]	0.23
	Age	1			Reference
		2	4.02	[1.26–17.9]	0.03 *
		≥3	2.86	[0.92–12.6]	0.1
PCR	Season	Spring			Reference
		Summer	1.52	[0.74–3.19]	0.26
		Autumn	2.61	[1.19–5.82]	0.02 *
		Winter	1.21	[0.65–2.32]	0.55
	Sex	Female			Reference
		Male	0.85	[0.52–1.38]	0.51
	Age	1			Reference
		2	2.21	[1.07–4.83]	0.04 *
		≥3	2.91	[1.47–6.16]	0.01 *
**Sampling Year 2020 (October 2020–September 2021)**					
**Method**	**Factor**	**Category**	**OR**	**[95% CI]**	***p*-Value**
HCT	Season	Spring			Reference
		Summer	0.32	[0.11–0.86]	0.03 *
		Autumn	0.85	[0.39–1.93]	0.7
		Winter	0.92	[0.42–2.06]	0.84
	Sex	Female			Reference
		Male	0.68	[0.35–1.28]	0.24
	Age	1			Reference
		2	0.92	[0.41–2.19]	0.85
		≥3	0.64	[0.29–1.48]	0.28
PCR	Season	Spring			Reference
		Summer	2.49	[1.31–4.8]	<0.01 *
		Autumn	1.28	[0.71–2.34]	0.41
		Winter	1.33	[0.73–2.42]	0.35
	Sex	Female			Reference
		Male	0.70	[0.44–1.1]	0.12
	Age	1			Reference
		2	3.30	[1.76–6.33]	<0.01 *
		≥3	2.72	[1.51–5.02]	<0.01 *

* indicates significant differences (*p* < 0.05) compared with the reference categories.

## Data Availability

The data presented in this study are available from the corresponding author upon reasonable request.
